# C-Reactive Protein Causes Blood Pressure Drop in Rabbits and Induces Intracellular Calcium Signaling

**DOI:** 10.3389/fimmu.2020.01978

**Published:** 2020-08-28

**Authors:** Christopher Bock, Birgit Vogt, Stephan Mattecka, Gülcan Yapici, Patrizia Brunner, Sandra Fimpel, Juliane K. Unger, Ahmed Sheriff

**Affiliations:** ^1^Division of Nephrology and Internal Intensive Care Medicine, Medical Department, Charité - Universitätsmedizin Berlin, Berlin, Germany; ^2^iAdsorb GmbH, Berlin, Germany; ^3^Pentracor GmbH, Hennigsdorf, Germany; ^4^Department of Experimental Medicine, Charité - Universitätsmedizin Berlin, Berlin, Germany; ^5^Division of Gastroenterology, Infectiology and Rheumatology, Medical Department, Charité - Universitätsmedizin Berlin, Berlin, Germany

**Keywords:** CRP, cardiovascular system, SIRS, adrenergic receptor, calcium transient, calcium signaling, eNOS

## Abstract

Systemic diseases characterized by elevated levels of C-reactive protein (CRP), such as sepsis or systemic inflammatory response syndrome, are usually associated with hardly controllable haemodynamic instability. We therefore investigated whether CRP itself influences blood pressure and heart rate. Immediately after intravenous injection of purified human CRP (3.5 mg CRP/kg body weight) into anesthetized rabbits, blood pressure dropped critically in all animals, while control animals injected with bovine serum albumin showed no response. Heart rate did not change in either group. Approaching this impact on a cellular level, we investigated the effect of CRP in cell lines expressing adrenoceptors (CHO-α1A and DU-145). CRP caused a Ca^2+^ signaling being dependent on the CRP dose. After complete activation of the adrenoceptors by agonists, CRP caused additional intracellular Ca^2+^ mobilization. We assume that CRP interacts with hitherto unknown structures on the surface of vital cells and thus interferes with the desensitization of adrenoceptors.

## Introduction

C-reactive protein (CRP), a circulating acute-phase plasma protein, increases very rapidly and at high serum concentrations in response to infection or tissue injury. It has a Ca^2+^-dependent binding specificity for phosphocholine, which is widely distributed in polysaccharides of pathogens and in cellular membranes (1). Thus, CRP is able to opsonize a number of pathogens as well as membranes of damaged and necrotic cells, facilitates their removal and thereby also mediates tissue damage by activating complement (2). It is commonly established both as a systemic and non-specific marker of inflammatory processes and as a risk factor and mediator of cardiac events (3–6). In addition, CRP has been shown to directly activate circulating leukocytes with subsequent release of mediators that promote destabilization of intravascular plaques, thereby increasing the risk of cardiovascular events (7).

While it has been shown that blood pressure, heart rate and catecholamines as well as adrenergic receptors can affect circulating CRP concentrations, a systemic influence of CRP has hardly been investigated so far. The use of beta-adrenoceptor (β-AR) blockers has been found to correlate with reduced circulating CRP levels, presumably due to a reduction in shear stress on endothelial cells of the blood vessels and thus a reduced inflammatory response (8–10). In contrast, chronic stimulation of β-ARs and catecholamines triggers an inflammatory response in hepatocytes and thus leads to an increase in circulating CRP (11, 12). Furthermore, chronic, moderate CRP levels are associated with general hypertension, increased heart rate and increased pulse pressure (13, 14). However, a direct influence of CRP on adrenergic receptors resulting in acute effects on the cardiovascular system have not yet been proven to be causal (13–15).

In the clinical situation, critically ill patients, suffering from e.g., sepsis or acute pancreatitis often develop systemic inflammatory response syndrome (SIRS) and are characterized by hardly controllable hemodynamic variables such as low mean arterial pressure (<70 mmHg) with subsequent reduced organ perfusion and additionally elevated CRP levels (16). Both parameters are independently associated with clinical outcome, mortality and disease severity (17–19).

We investigated acute effects of infused CRP on the circulatory system *in vivo*. We chose healthy rabbits, which express comparable endogenous CRP levels as humans (rabbits: <3 mg/L baseline, >100 mg/L acute phase; humans: <1 mg/L baseline, >500 mg/L acute phase) as animal model because the binding activity of rabbit CRP is comparable to that of human CRP and both play a similar biological role in the innate immune system (20, 21). Furthermore, we investigated the influence of CRP on signal transduction *in vitro*. We used cell models with expression of α- and β-adrenergic receptors, which belong to the group of G protein-coupled receptors (GPCRs) but may have different pathways of signal transduction. Activation of α_1_-adrenergic receptors results in a Ca^2+^ release from the intracellular Ca^2+^ storage in the endoplasmic reticulum. The activation of β_1_- and β_2_-ARs leads to the opening of voltage-gated Ca^2+^ channels and thus to an influx of external Ca^2+^ across the plasma membrane. Changes in the intracellular free Ca^2+^ concentration can be observed and quantified by ratiometric fluorescence (22).

Ca^2+^ as an essential second messenger in intracellular signal transduction pathways plays a key role in the control of various cellular functions such as contraction, proliferation and transcription (23). For the first time, we report a direct effect of CRP on the Ca^2+^-signaling in two different cell lines.

## Animals, Materials, and Methods

### Preparation of Purified Human CRP

Human plasma samples were collected from patients with high CRP concentrations, pooled and stored frozen at −20°C until the experiments began. Plasma pool was thawed, and CRP was purified by running an affinity chromatography column of a selective CRP-binding matrix (Pentracor GmbH, Germany) (24). Elution of CRP was performed by an elution buffer consisting of 0.1 M Tris, 0.2 M NaCl, and 2 mM EDTA, pH 8, which inhibited the Ca^2+^-dependent binding of CRP to the matrix. The wash fractions were collected. After determining the CRP concentration via ELISA, the fractions were centrifuged over a molecular cut-off filter of 50 kDa in order to concentrate pentameric CRP and to exchange the buffer system. CRP was then diluted in application buffer (NaCl 0.9%, Tris 10 μM, EDTA 20 μM, Pluronic 1 g/L) to a final concentration of 15 mg/mL. In order to keep possible endotoxin contamination as low as possible, we used a sterile CRP adsorber, treated the devices with NaOH or depyrogenated the glass devices at 220°C, prepared everything with sterile cell culture water and used sterile endotoxin free disposable material. Before elution we washed several times. After elution we concentrated the CRP by ultracentrifugation and collected it in fresh sterile buffer.

### Animals

Seven female New Zealand White rabbits were purchased from Harlan Winkelmann with either 3 kg or at 8–10 weeks age. At the time point of the experiment, 3 animals were ~24 weeks old (4.3–5.3 kg) and 4 animals were ~10 weeks old (1.85–1.95 kg). All rabbits were housed in groups of five and according to FELASA guidelines. Health monitoring at the commercial breeder followed the FELASA recommendations. The study protocol (Reg. No.: G0299/09) was approved by the University Animal Care Committee and the federal authorities for animal research in Berlin, Germany.

### Injection of CRP in Rabbits (*in vivo*)

On the day of the experiment, rabbits were anesthetized intramuscularly with a mixture of 35 mg/kg ketamine (Ursotamin, Serumwerk Bernburg, Germany), 0.25 mg/kg medetomidine (Domitor, Pfizer, USA) and 0.1 mg/kg atropine (Atropinsulfat B.Braun, B.Braun, Germany), followed by inhalation of 1.1 Vol.% isoflurane (Forene, Abbott, USA) via mask. This was according to standard practice guidelines (25). A catheter (Vygonule, B. Braun, Germany) was inserted in an ear artery for continuous measurement of the mean arterial blood pressure (ABP) and the heart rate (HR) using the monitoring system HP Model 66S. Data points were recorded every 20 ms. For later analysis of ABP and HR the analog output-signal of the monitor was transformed into digital signals (HB627, H-Tronic, Germany). These were converted and saved with a curve performance software (RealView3.0, Abacom, Germany). The RealView data were transferred to Excel (Microsoft Office Software) for further processing.

Another catheter was inserted in an ear vein for administration of either CRP (3.5 mg/kg body weight corresponding to 50 μg/mL blood volume) or bovine serum albumin (BSA) as control. CRP or BSA, each diluted in application buffer (15 mg/mL), were slowly injected intravenously (volume ranging from 0.44 to 1.17 mL over 1 min) followed by a flush of 0.9% saline. Seven animals received treatment with CRP and five of these received treatment with BSA as a control protein either 1 h after or before CRP application. To rule out individual animal effects, control and CRP application was performed and compared in the same rabbits. Blood samples were taken before and after the experiment.

The first animal died unexpectedly and immediately after the intravenous injection of CRP. It is to be assumed that the administration was too fast. The following rabbits were injected more slowly.

### ELISA for Human CRP

Rabbit blood samples were collected in serum tubes. Serum was separated with centrifugation and frozen for later analysis. A 96-well-ELISA-plate (Corning, USA) was coated with 9 μg/mL polyclonal rabbit-anti-human CRP-antibody (Dako, Denmark). After incubation, wells were washed (0.1% Tween in PBS) and blocked with a 0.1% casein-solution. Samples were diluted in 5 mM EDTA-solution and incubated in coated wells. Horseradish-peroxidase-conjugated rabbit-anti-humanCRP antibodies (14 ng/mL) were added. After addition and incubation of peroxidase substrate (tetramethylbenzidine, SigmaAldrich, USA) reaction was stopped by 1 M sulphuric acid. Finally, photometric measurement followed at 450 nm and 620 nm (reference) with an ELISA-reader (Sunrise, Tecan, Switzerland). All measurements were performed at least twice with triplicates.

### Intracellular Ca^2+^-Signaling Measurements (*in vitro*)

For the assays on Ca^2+^ signaling we used two different cell lines. CHO-α1A is a clonal cell line derived from Chinese hamster ovaries stably exhibiting overexpression of the human α_1A_-adrenergic receptor (α_1A_-AR) with efficient coupling to intracellular calcium (${\text{Ca}}_{\text{i}}^{2+}$). DU-145 is a clonal tumor cell line derived from human prostate cancer that expresses different GPCRs such as α_1_-AR and β-ARs. CHO-α1A cells were cultivated in Ham's F12 medium supplemented with glutamine and DU-145 cells in RPMI-1640 medium, respectively, with the addition of 10% fetal calf serum (FCS) and 1% penicillin/streptomycin solution at 37°C and 5% CO_2_ saturation. CHO-α1A and DU-145 cell lines were kindly provided by Dr. Gerd Wallukat, former Max Delbrueck Center for Molecular Medicine, Berlin.

To observe and quantify the real-time changes of cytosolic Ca^2+^ alterations the fluorescent dye Fura-2 and a Victor X2 fluorescence multiplate reader (Perkin Elmer, Langenfeld, Germany) equipped with an injector unit were used. In light diminished conditions, cells were treated with 5 μM Fura-2 (Merck Biosciences, Bad Soden, Germany) for 45 min at 37°C. Cells were washed and incubated in HBSS-HEPES (Hanks' Balanced Salt Solution modified with 10 mM HEPES) plus 2,5 mM Probenecid for another 30 min before transfer to the microplate reader (26). Real-time fluorescence measurements were recorded every 1.65 s over a period of 150 s at an alternating excitation wavelength of 340 nm and 380 nm and emission wavelength of 510 nm. The intracellular free Ca^2+^ concentration can be obtained from the ratio of emission after stimulation at both wavelengths. In order to compare the Ca^2+^ signals of all experiments, the baseline values were subtracted and the difference value (Δ${\text{Ca}}_{\text{i}}^{2+}$) was plotted against time.

To determine the effect of CRP in comparison to that of pharmacological adrenergic receptor agonists, we used phenylephrine (PE), a selective α1-AR agonist and isoprenaline (isoproterenol, ISO), a non-selective β-AR agonist. At the beginning, the baseline value was measured and after 30 s CRP, PE or ISO was added. All conditions were performed in technical duplicates.

## Results

### Experiment 1: Rapid Drop of Blood Pressure in Rabbits After CRP Injection (*in vivo*)

To determine whether hCRP has a direct effect on the resting heart rate and blood pressure of rabbits, animals were injected with hCRP (3.5 mg/kg) without further treatment and the HR and ABP were monitored over time. At the beginning and end of each experiment, blood samples were collected and analyzed by a specific human CRP ELISA. No human CRP was detectable in rabbits prior to CRP injection (data not shown). About 1 h after injection, the mean circulating human CRP concentration was 45 ± 9.1 mg/L (range of 7 animals: 35 to 63 mg/L).

**Figure 1:**
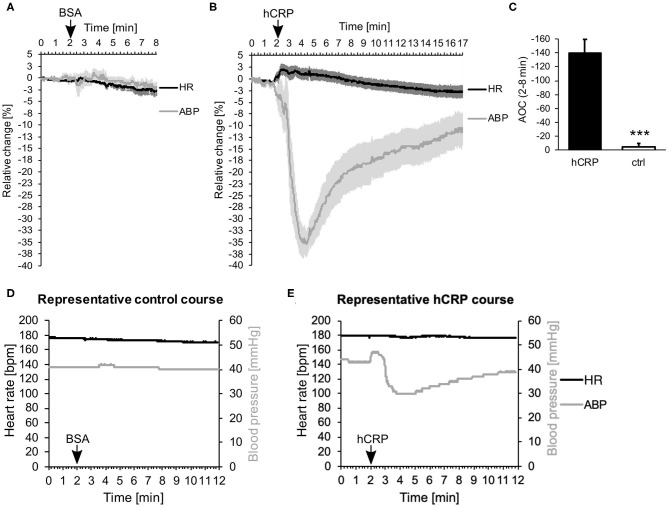
**(A–E)** Human CRP injection triggers rapid drop in blood pressure in rabbits. **(A,B)** 5 **(A)** or 7 **(B)** rabbits were monitored for their mean arterial blood pressure (ABP) and heart rate (HR). Data points were recorded every 20 ms and are plotted for every second. After 2 min, 3.5 mg/kg hCRP or BSA as control were injected intravenously. Graphs depict relative change in mean ABP (gray) and HR (black) over time ± SEM (standard error of the mean) as lighter shade envelope. The difference between placebo (BSA) and CRP-infusion group is highly significant (Fisher's exact test: ***p < 0.001). **(C)** Calculation of the area between the curve of ABP and the zero line (Area Over the Curve = AOC) depicted in **(A,B)** between 2 and 8 min. Mean area of 7 (hCRP) or 5 (ctrl) animals ± SEM. Control area is significantly smaller than hCRP treated area. *** < 0.001 after Student's *t*-test. **(D,E)** Representative animals from the control group **(D)** or the hCRP group **(E)** with absolute values of HR (black) and ABP (gray) over the time course of 12 min.

Immediately after intravenous injection of CRP, we observed a surprisingly drastic drop in ABP, while the HR stayed the same (Figure 1A). Control animals, which were injected with the same amount of BSA solution, showed no changes in ABP or HR (Figure 1B). Figure 1C compares the effect shown in Figures 1A,B as area between the curve and the zero line. Within the first 6 min after i.v. injection the area was significantly larger in animals treated with CRP compared to control animals. Representative courses of both groups are depicted in Figures 1D,E and show the absolute values of ABP and HR. Here, the representative animal injected with CRP exhibited a drop in blood pressure from 54 to 30 mmHg at its lowest point (at 4 min = 2 min after injection), which did not recover within 10 min after injection, while the HR did not counteract at all as would be normally expected.

The experimental setup as such, including the anesthesia and the catheters used, had no effect on the heart rate and lowered blood pressure only slightly over time (Supplemental Figure 1).

### Experiment 2: Characterization of CRP-Induced Calcium-Signaling in Epithelial Cells (*in vitro*)

Our next step was to investigate the influence of CRP on adrenoceptor signaling *in vitro*. As part of the group of GPCRs, these receptors have different pathways of signal transduction, which all use Ca^2+^ as second messenger. We therefore examined whether CRP affects calcium influx *in vitro* in two different cell lines expressing either exclusively α_1A_-AR (CHO-α1A) or both α and β-ARs (DU-145).

In the first assay, the effect of CRP on the intracellular free calcium concentration ([Ca^2+^]_i_) was investigated in comparison to the adrenoceptor stimulation by agonists. The maximum effective concentration of PE was 5 μM and of ISO 1 μM, respectively. CRP was applied to reach a final concentration of 50 μg/mL. As control protein, we used human serum IgG (50 μg/mL).

**Figure 2:**
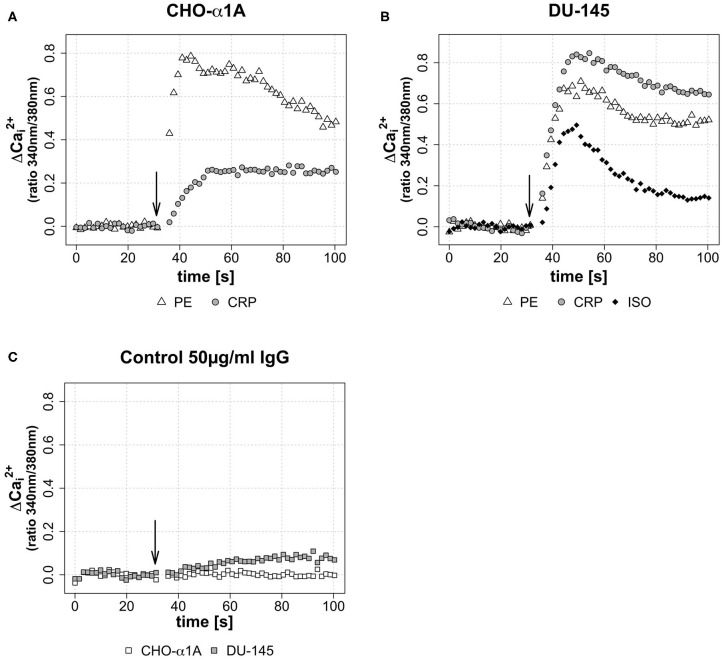
**(A–C)** Change of intracellular calcium concentration ([Ca^2+^]_i_) after CRP application, depicted as Δ${\text{Ca}}_{\text{i}}^{2+}$ over the time. **(A–C)** Arrow indicates application of either CRP (final concentration 50 μg/ml), control IgG (final concentration 50 μg/ml), phenylephrine (PE, final concentration 5 μM) or isoprenaline (ISO, final concentration 1 μM). Graphs depict measured ratio 340/380 nm every 1.65 s. Data are representative of three experiments. **(A)** In CHO-α1A cells, Δ${\text{Ca}}_{\text{i}}^{2+}$ rises 20 s after CRP application to a maximum of approximately 0.27, followed by a persistent plateau. For comparison: the application of α_1_-AR agonist PE leads after 10 s to a Δ${\text{Ca}}_{\text{i}}^{2+}$ maximum of ~0.78, followed by a decrease. **(B)** In DU-145 cells, 18 s after CRP application Δ${\text{Ca}}_{\text{i}}^{2+}$ reaches a maximum of approximately 0.83. PE and the β1-AR agonist ISO lead to a Δ${\text{Ca}}_{\text{i}}^{2+}$ maximum of ~0.67 after 15 s (PE) and ~0.48 after 18 s (ISO), respectively. **(C)** No (CHO-α1A) or only a slight (DU-145) increase was observed after application of human serum IgG as control protein.

Figures 2A,B illustrate CRP-induced increase in [Ca^2+^]_i_ in CHO-α1A and DU-145 cells compared with the full adrenoceptor agonist effect of PE and ISO. In both cell lines, CRP elicited increases in [Ca^2+^]_i_, consisting of an initial transient phase followed by a sustained phase. In CHO-α1A cells, CRP-induced [Ca^2+^]_i_ showed a two-thirds lower and slower increase compared to PE, but then remained stable over the entire measurement period. In DU-145 cells, the increase of [Ca^2+^]_i_ after CRP application was higher than after PE and ISO, and approximately as fast. The subsequent maintenance phase decreased slowly, comparable to the effect of PE. The application of human serum control IgG had little or no effect on [Ca^2+^]_i_ in both cell lines (Figure 2C).

**Figure 3:**
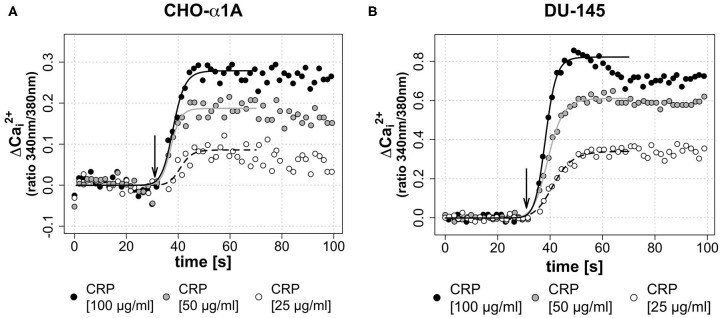
**(A,B)** Dose-dependent effect of CRP on [Ca^2+^]_i_. In both cell lines, a higher concentration of the applied CRP leads to a faster and higher increase of [Ca^2+^]_i_. **(A,B)** Arrow indicates application of CRP. Graphs depict measured ratio 340/380 nm every 1.65 s. Data are representative of three experiments. A 4-parameter sigmoidal logistic fit curve was fit to demonstrate the typical dose response kinetics. **(A)** In CHO-α1A cells, CRP was applied at different final concentrations (25, 50, or 100 μg/ml). The maximum Δ${\text{Ca}}_{\text{i}}^{2+}$ was 0.08 for 25 μg/ml CRP, 0.18 for 50 μg/ml CRP and 0.28 for 100 μg/ml CRP. The half-maximum Ca^2+^ signal, which corresponds to the inflection point of the logistic regression curve, was reached after 10.9/9.3/8.1 s (CRP 25/50/100 μg/ml). **(B)** In DU-145 cells, CRP was applied at different final concentrations (25, 50 or 100 μg/ml). The maximum Δ${\text{Ca}}_{\text{i}}^{2+}$ was 0.34 for 25 μg/ml CRP, 0.61 for 50 μg/ml CRP and 0.82 for 100 μg/ml CRP. The half-maximum Ca^2+^ signal was reached after 11.3/9.3/8.2 s (CRP 25/50/100 μg/ml).

To investigate a dose-dependent effect of CRP on [Ca^2+^]_i_, in a second assay CRP was applied in three different final concentrations (25, 50, and 100 μg/mL). Figures 3A,B demonstrate that the CRP-induced increase of [Ca^2+^]_i_ in both cell lines was depending on the CRP concentration. A high CRP concentration caused a faster and higher [Ca^2+^]_i_ increase.

**Figure 4:**
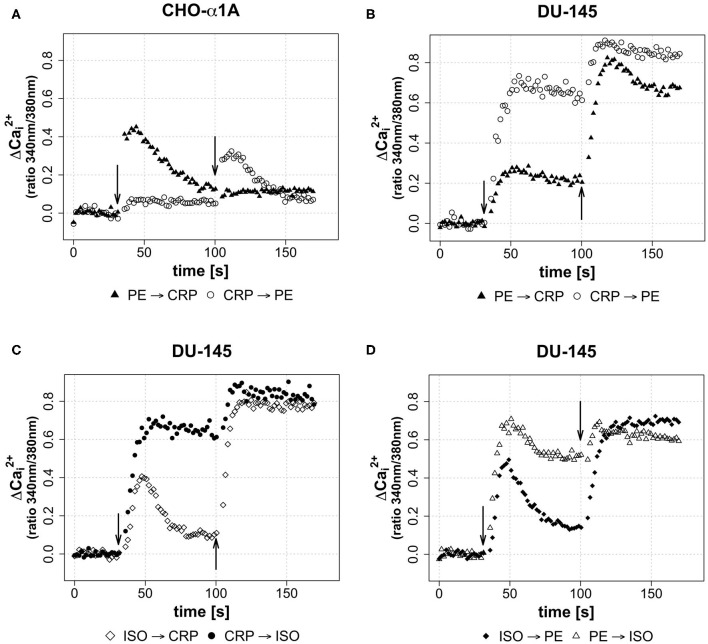
**(A–D)** Effect of sequential application of PE or ISO and CRP on [Ca^2+^]_i_. **(A–D)** Arrows indicate different applications of either CRP (final concentration 50 μg/ml), phenylephrine (PE, final concentration 5 μM) or isoprenaline (ISO, final concentration 1 μM) in sequential combinations apparent from legends. 2nd application was performed after 100 s of measurement, thus 70 s after 1st application. Graphs depict measured ratio 340/380 nm every 1.65 s. Data are representative of three experiments.

In a third assay, the effect of different sequential applications of CRP and adrenoceptor agonists on [Ca^2+^]_i_ was investigated. Therefore, either CRP (50 μg/mL) was added 70 s after full adrenoceptor stimulation with PE or ISO or the agonists were added after CRP stimulation. This was performed in order to gain insight whether the CRP induced calcium influx is triggered via adrenoceptor activation or whether it uses independent mechanisms. Under the influence of an agonist, a receptor is desensitized in order to avoid overstimulation of a cell by signal molecules and no further [Ca^2+^]_i_ increase was expected. The effect of sequential application of CRP following PE/ISO or *vice versa* is shown in Figure 4 for both cell lines. In CHO-α1A cells, the application of CRP following PE did not lead to a further increase of [Ca^2+^]_i_, but the decline of [Ca^2+^]_i_ was stopped and stayed upregulated on a steady level, while the stimulation with PE after CRP application additionally increased [Ca^2+^]_i_ (Figure 4A)_._ In DU-145 cells, CRP following PE additionally mobilized Ca^2+^, and PE following CRP also triggered an additional increase of [Ca^2+^]_i_ (Figure 4B). When CRP was given after ISO, the increase in [Ca^2+^]_i_ was even higher and remained on a sustained plateau, whereas application of ISO after CRP also enhanced [Ca^2+^]_i_ but only slightly (Figure 4C).

Last, we wanted to see whether the combination of the two different agonists inducing complete adrenoceptor activation of their respective adrenoceptor increases [Ca^2+^]_i._ Stimulation of DU-145 cells with ISO led to a brief increase in [Ca^2+^]_i_, which was followed by a decrease and a strong increase after subsequent PE application. *Vice versa* application differed, as the PE-induced increase stayed upregulated and was again enhanced slightly by ISO stimulation (Figure 4D).

## Discussion

The study was designed to investigate a possible influence of CRP *in vivo* on a healthy organism, respectively, *in vitro* on vital cell lines that are neither damaged, necrotic nor apoptotic. To prevent possible side effects of commercial CRP solutions (27) we purified CRP by ourselves from pooled frozen human plasma. The purified native human CRP is almost completely present in its natural pentameric form.

The intravenous injection of CRP in rabbits has a minimal direct influence on the heart rate (HR) but a dramatic effect on the arterial blood pressure (ABP) as shown in Figure 1. The intravenous administration of CRP in anesthetized rabbits resulted in an immediate drop in blood pressure, while HR remained the same and did not counteract as we would expect. Normally, a drop in blood pressure would lead to an activation of the sympathetic nervous system by suppressing the baroreceptor reflex and thus to an increase in heart rate. The physiological mechanisms behind our observations are therefore not clear. It took about 15 min for ABP to return to baseline.

Despite all caution in the first animal, the intravenous injection of CRP was probably too fast. The animal died immediately, we suspect due to a drastic drop in blood pressure caused by the effect of CRP. The administration of pure solution buffer containing bovine serum albumin (BSA) as a protein control showed no effect.

Previous studies on the direct effects of human CRP on the cardiovascular system *in vivo* recorded changes after a few minutes at the earliest. In rats, for example, the administration of 20 mg/kg CRP had no effect on blood pressure compared to control animals, but ABP was measured only every 15 min after administration (28). In contrast, we measured the acute effect of CRP administration at very small intervals right from the time of injection. Since the observed effect in rabbits reached its peak about 3 min after CRP injection and then declined back to baseline, measurements after 15 min would probably not have shown any changes.

Another study, directly administering recombinant CRP into human subjects, also showed no changes in hemodynamic parameters and vasodilator capacity (29). However, CRP serum levels increased to only 30 mg/L after infusion. Further, hemodynamic parameters and vascular reactivity were measured 6 h after CRP infusion not accounting for acute effects, but rather inflammatory-mediated mechanisms. Also, in a study by Bisoendial et al., in which healthy human volunteers were given recombinant CRP, the CRP level increased only up to 23.9 mg/L and inflammatory blood parameters were determined at the earliest after 1 h (30). Nevertheless, they observed a systemic inflammatory response and stimulation of the coagulation cascade by administration of CRP.

Lane et al. similarly investigated the effects of a single intravenous bolus of natural human CRP, but in seven volunteers over a period of 0.5 h to 10 days (31). They found no clinically significant changes, neither physical nor hematological. In contrast, we investigated the effect of human CRP on rabbits, which differ significantly from humans in both body size and metabolic rate. In addition, the rabbits were anesthetized while the human probands were not. The drastic immediate effect, which started 2 min after the infusion, could be due to a faster availability of the infused CRP in a smaller organism.

To our knowledge, this study is the first to demonstrate a direct and acute effect of CRP on blood pressure *in vivo*, which could account for the hardly controllable hemodynamic parameters of patients with excessive CRP levels and hint toward a molecular interaction of CRP with the adrenergic system.

Subsequently, we investigated *in vitro* whether CRP affects adrenergic receptors, since these are mainly found on smooth muscle cells of the blood vessels and on cardiomyocytes and are thus significantly involved in the regulation of blood pressure. CHO-α1A cells are transfected epithelial cells that express only α_1A_-AR (32). DU-145 prostate tumor cells express a number of different GPCRs such as α_1−_, β_1−_, and β_2_-ARs and H1-histamine receptors (33, 34). Both cell lines show efficient coupling to Ca^2+^ of the adrenoceptors and can therefore be used for investigation of this signal transduction pathway.

We observed that CRP triggered calcium signaling in both cell lines (Figure 2). This increase in [Ca^2+^]_i_ was dose-dependent, i.e., the higher the applied CRP concentration, the more and faster Ca^2+^ was mobilized in the cell (Figure 3). To our knowledge, this is the first time to show a direct effect of CRP on cellular calcium signaling.

Further, the CRP induced calcium influx was comparable in intensity and duration to the stimulation with the adrenoceptor agonists PE and ISO.

In order to investigate which molecular mechanism CRP uses to directly affect calcium levels, we sequentially applied adrenoceptor agonists and CRP in different combinations. After desensitizing α-AR or β-ARs with PE and ISO, respectively, CRP still triggered an additional calcium increase in DU-145 cells (Figures 4B,C). This was not the case in CHO-α1A cells, in which after PE stimulation the declining calcium signal did not rise again with the CRP stimulus but only remained on a plateau (Figure 4A). *Vice versa*, both agonists additionally mobilized calcium after the CRP induced calcium rise in both cell lines.

Interestingly, after desensitization of adrenoceptors, CRP managed to increase intracellular calcium levels more dramatically than PE or ISO in DU-145 cells (Figure 4D). This hints toward a molecular mechanism independent of adrenoceptor activation. On the other hand, in CHO-α1A cells, CRP did not trigger calcium influx after desensitization of α-ARs, which in this cell line is the only expressed adrenoceptor. Potentially, CRP activates all types of adrenoceptors, and can therefore not trigger more calcium signaling in CHO-α1A cells, as no β-ARs are expressed.

The well-established function of CRP as protein of the innate immune system is the elimination of cells via activation of complement triggered by binding of CRP to special phosphocholine components. Phosphocholine is e.g., accessible to CRP on the surface of pathogens (C-Polysaccharide) or in the plasma membrane of damaged or necrotic cells, which exhibit lysophosphatidylcholine (35, 36). We show that, additionally, CRP seems to be able to act on healthy cells and increase intracellular free Ca^2+^. The underlying molecular mechanism for that is unclear and using different combinations of adrenoceptor agonists and CRP stimulation gave preliminary results, which hint toward an adrenoceptor-independent pathway. However, it is still possible that CRP engages adrenoceptors directly as the calcium influx kinetics resemble those triggered by adrenoceptor agonists. The additive effect of CRP on PE and ISO could potentially either be explained by activating the saturated adrenoceptors differently than the agonists or by the hypothesis that CRP engages both α- and β-ARs, while the agonists were specific for only one type.

Further, CRP could interfere with their desensitization, perhaps it might even affect other GPCRs. The mechanism remains obscure and further research should follow to understand this effect in detail.

Several *in vitro* studies on endothelial cells of different species suggest a damaging effect of CRP on the cardiovascular system by influencing the endothelial nitric oxide (NO) synthase (eNOS) in expression and function. CRP uncouples eNOS, resulting in increased superoxide production and reduced NO synthesis, which can lead to vasodilatation disorders (37–41). eNOS is activated by an increase in the [Ca^2+^]_i_ and the association of a Ca^2+^/calmodulin complex with the enzyme (42). Thus, endothelial NO production is enhanced which induces vasodilatation in the organism (43). Our *in vitro* findings appear to be reflected by the drastic drop of blood pressure after CRP injection in our *in vivo* experiments. By a hitherto undiscovered mechanism, CRP induces Ca^2+^ signaling, which may have led to vasodilatation due to increased NO production via activation of eNOS. This seems to outweigh the eNOS uncoupling effect of CRP. There might be a dose dependence. Further studies focusing on the impact of CRP on the NO system might be promising.

## Conclusion

The findings of our animal experiments suggest a direct systemic influence of CRP in rabbits *in vivo*, resulting in a drastic drop of blood pressure shortly after the intravenous injection. The organism did not counteract this effect by upregulating the heart rate as expected. On the cellular level, a corresponding effect can be found. The application of CRP modulates the calcium signaling to a dose-dependent extent. The increase in intracellular calcium concentration triggered by CRP is potentially mediated by adrenoceptors, but could also use a different molecular mechanism.

## Study Limitations

In order to obtain a first indication if CRP has a direct effect on the circulatory system of rabbits *in vivo*, only few animals were used in the experiments for ethical reasons. Further, although the biological role and properties of CRP are comparable, other species differences could explain why this effect has not been demonstrated in humans so far. The *in vitro* experiments have been performed with transfected cells (CHO-α1A) or with cells not yet precisely characterized in regard to GPCRs (DU-145). It remains to be investigated if the observations can also be reflected in humans.

## Data Availability Statement

The raw data supporting the conclusions of this article will be made available by the authors, without undue reservation.

## Ethics Statement

The study protocol (Reg. No. G0299/09) was approved by the University Animal Care Committee and the federal authorities for animal research in Berlin, Germany.

## Author Contributions

CB, BV, GY, JU, and AS designed and performed the experiments. CB, SM, PB, SF, and AS evaluated the data and created the manuscript. All authors contributed to the article and approved the submitted version.

## Conflict of Interest

SF and PB were employed by Pentracor GmbH and iAdsorb GmbH, respectively. The remaining authors declare that the research was conducted in the absence of any commercial or financial relationships that could be construed as a potential conflict of interest.
