# Supplementary figures and images for: C-Reactive Protein Causes Blood Pressure Drop in Rabbits and Induces Intracellular Calcium Signaling
Source: Front Immunol. 2020 Aug 28;11:1978. doi: 10.3389/fimmu.2020.01978 (PMC7483553; doi:10.3389/fimmu.2020.01978)

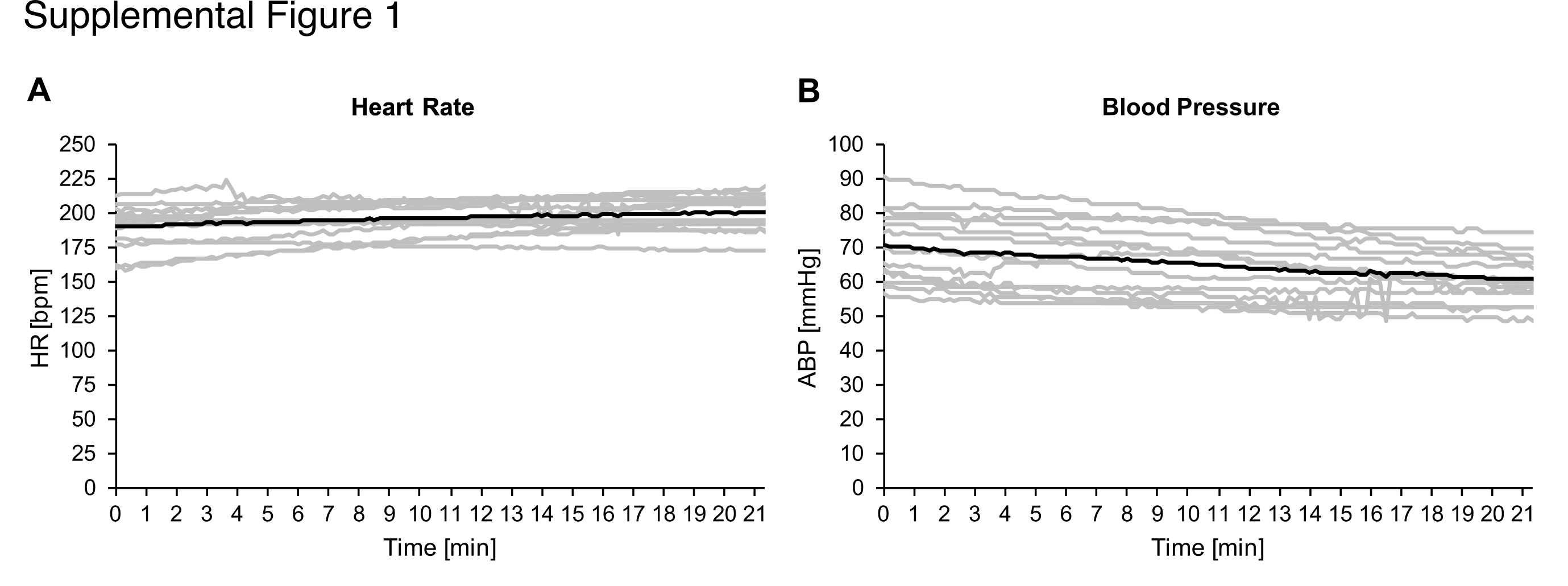

Supplement: Supplementary file 1 [file Image_1.jpg]
